# Popular Science Department

**Published:** 1882-12

**Authors:** 


### POPULAR SCIENCE DEPARTMENT.

### WHAT THE CELL CAN DO.

Water, earth salts, and the gases—the raw materials which the plants suck up—are changed within the cells into starch and sugar, gum and woody fiber, albumen and wax, oil and resin, into powerful medicines and deadly poisons. The simplest plant possesses an art which the most skillful chemist has not been able to learn from it. It is true that the chemist can artificially prepare in his laboratory many of the substances which the plant-cell likewise produces ; he can convert the starch of the potato into the sugar that gives the wine-grape it sweetness ; this, again, he can transform into the fruit-acids which, in connection with the sugar, give the berries their fresh and agreeable taste ; he can even produce the flavor of the fruits from the fusel-oil which he obtains by the fermentation of the sugar. He can make the oil of bitter almonds from benzoic and formic acids ; he can, with as good art, imitate the pungent taste of the pepper, and the biting one of the mustard-seed, and the narcotic poison which only the nightshade has hitherto prepared for the healing of sore eyes. He can produce from the sap of firs the crystal-needles of the vanilla, for which a Mexican orchid has heretofore been obliged to give up its pods ; from the distillation of wood he obtains a smoky fluid, from which he procures salicylic acid, for the production of which the flowers of the meadow-sweet or the bark-tissues of the willow were formerly required ; and from this he makes also the ink-coloring gallic acid, which formerly only a little wasp knew how to draw out by its sting from the cells of the oak, and the aroma of the wood-ruff. He has made the work of the cells in the madder-root superfluous, for he has fabricated its costly dyes, along with a hundred other splendid pigments, out of tar oil and stone-coal ; and is now on the point of taking its works away from the indigo-plant by artificially producing indigo. But a raw material which has at some time been brought forth out of the laboratory of a living plant-cell always lies at the foundation of all these manipulations of the chemist, wonderful as they are. And, notwithstanding the immense progress that modern chemistry has made within the last ten years, its art is still limited at this point : no prospect yet exists that it will be able, artificially, to produce the most important of all the substances that go to build up the bodies of animals and plants, and to form their living cell-tissues—protoplasm, or the envelope of the plant-cells, the matter of the muscles and nerves.—PROFESSOR FERDINAND COHN, *in Popular Science Monthly for December.*

### REGARD OF THE ANCIENTS FOR ANIMALS.

In ancient Egypt, when a cat died in the house, the inhabitants shaved their eyebrows ; if a dog died, they shaved their whole body.

In Athens, one of the laws of Triptolemus declared that no one had a right to inflict a wrong upon a living creature. The Greeks were aware of the tender and affectionate care which the young of the stork exhibited for their old parents, and recorded that, when the latter lost their feathers from age, the young stripped themselves of their down for them and fed them with the food they collected. This was the origin of the Greek law called "the law of the stork," by virtue of which children were obligated to take care of their aged parents, and those who refused to do so were declared infamous. How different is it in our modern societies! Pierquin remarks with reason that, as man rises, he treats animals as if they were correspondingly degraded. For a long time they had the same rights. During the middle ages they were allowed a part in religious ceremonies. At Milan they figured in the festivals of the kings; and processions of animals appear in the bas reliefs of the cathedrals of Strasburg, Mans, and Vienne (Isere). On Holy Wednesday all the clergy of the church of Rheims went to Saint Remi to make a station there; the canons, proceded by the cross, were arranged in two lines, each drawing a herring after him with a cord; and each one was intent upon saving his own fish, and stepping upon that of the canon in front of him (Anquetil, "Histoire de Reims"). At Paris, the procession of the fox was as much enjoyed as the festival of the ass. The animal, dressed in a kind of surplice, wearing the mitre, had his place in the midst of the clergy: a fowl was put within his reach; he often forgot his pious functions to spring upon the bird and devour it in the presence of the faithful. Philip the Fair was very fond of this procession (Sanval, "Antiquities de Paris"). Only a few years ago, the procession of the fat ox remained, a survival from the pagan feasts, a real piece of wreckage from vanished civilizations.

While the rights of animals were thus recognized, their duties toward man did not escape the earlier legislators, who severely punished their crimes and attempts upon human life. The law of Moses (Exodus xxi, 28, 29) recites: "If an ox gore a man or a woman that they die; then the ox shall be surely stoned, and his flesh shall not be eaten; but the owner of the ox shall be quit. But if the ox were wont to push with his horn in time past, and it hath been testified to his owner, and he hath not kept him in, but that he hath killed a man or a woman; the ox shall be stoned, and his owner also shall be put to death."

Judgments based on this principle are recorded at Athens and Rome. According to Pierquin, Democritus wished an animal, which had occasioned some major damage, to be punished with death. Under Domitian, according to the report of Martial, the ingratitude of a lion toward its mater was severely punished. Columella and Varro say that the ancient Romans regarded the ox as the companion of the labors of man, and that the act of killing one was regarded as a homicide and punished in the same way; and the ox enjoyed the same privilege in Attica and Peloponnesus. It is also said that the Arabs in the mountains of Africa formerly crucified lions, guilty of murders, upon trees, as warnings to others.—A. LACASSAGNE, *in Popular Science Monthly for December.*

